# Gastric ultrasound for feeding intolerance in the ICU: close but not quite right

**DOI:** 10.1186/s12876-022-02104-4

**Published:** 2022-01-25

**Authors:** Lynn Vernieuwe, An Wallyn, Lisa Van Dyck, Peter Van de Putte

**Affiliations:** 1 Department of Anaesthesiology, AZ Klina, Augustijnslei 100, 2930 Brasschaat, Belgium; 2 Department of Anaesthesiology, Imeldaziekenhuis, Imeldalaan 9, 2820 Bonheiden, Belgium; 3 Department of Anaesthesiology, UZ Leuven, Herestraat, 3000 Leuven, Belgium

## Abstract

In the January 2021 issue of BMC Gastroenterology, Elmokadem et al. report their findings on the use of gastric ultrasound (GUS) for the evaluation of feeding intolerance—defined as high gastric residual volume—in critically ill patients. We voice in this correspondence our concerns regarding certain methodological flaws and believe the study results should be interpreted with caution. The authors applied a model unvalidated for non-clear fluids as enteral feeding, the scanning protocol was not clearly described and essential anatomical landmarks required for correct interpretation are not visible in the presented images. Additionally, since GUS was not compared to a gold standard, we believe the authors’ conclusion may be overoptimistic and does not undoubtedly answer the primary outcome.

To the editor,

We read with interest the article in *BMC Gastroenterology* that evaluated the efficacy and safety of itopride in feeding intolerance of critically ill patients receiving enteral nutrition [1]. The authors used gastric ultrasound (GUS) to assess gastric contents and gastric residual volumes (GRVs) in ICU patients, whereby enteral feeding intolerance was diagnosed when the GRV exceeded 250 ml at any time. Feeding intolerance is an important problem in the ICU population and we congratulate the authors for their contributions. However, we are concerned about the methodology regarding the use of GUS for GRV measurements—and therefore believe the results of this study should be interpreted with caution—for the reasons given below.

First, the authors used a mathematical model for calculating total gastric fluid volumes that has been validated in different patient populations but for *clear* fluids only. The model as used by the authors has *not* been validated for estimating non-clear fluid volumes such as enteral feeding solutions which might possibly lead to serious under- or overestimation of total gastric fluid volumes [2].

Second, the figures as provided of the performed exams seem to be incorrect. It is mandatory that specific important anatomical landmarks are visualized simultaneously to assure one is scanning the antrum of the stomach when measuring its cross-sectional area: these are the left lobe of the liver and the aorta [2]. We were unable to identify the aorta in the images provided in the article. Moreover, the presence of the gallbladder in Figure 2 C while scanning the antrum is extremely atypical and suggests another gastrointestinal structure than the antrum was scanned (possibly duodenum).

Third, we are missing some information in the methodology, which we believe is essential to fully understand how GUS was performed in this study. There is no information provided about the experience of the physician scanning the patients nor the number of measurements performed per exam per patient. The prediction model used for measuring GRV is only applicable when a similar scanning protocol to that used in the original study is followed [3]. It demands three measurements of antral cross-sectional area of which the average is taken. From the description of the methodology in the article, it is unclear whether this was the case. Likewise, it is unclear whether measurements were directly taken at the bedside, or merely images were obtained on which measurements were performed at a later time, perhaps by a different examiner.

Fourth, ultrasound was included in the paper as a tool for patient inclusion as well as a guide in continuing nutritional therapy in addition to being the primary outcome of the study, which in our view is conflicting.

Last, the authors conclude that gastric ultrasound offers ‘reliable assessments of fluid volumes in the GE modality [1]. Since there was no actual comparison with a gold standard or other measurement technique and in the spirit of the first remark, this conclusion may be overoptimistic in our opinion and does not undoubtedly answer the primary outcome.

Hence, we think that the results of this study should be interpreted in light of these concerns.

## Data Availability

Not applicable.

## Abbreviations

GUS: Gastric ultrasound
GRV: Gastric residual volume
ICU: Intensive Care Unit
